# Chitosan Coatings with Essential Oil of *Schinus lentiscifolia* Marchand for the Management of Blue Mold and Preservation of Postharvest Quality of ‘Fuji’ Apples

**DOI:** 10.3390/foods15112023

**Published:** 2026-06-04

**Authors:** André Rodrigues da Costa, Roseli Lopes da Costa Bortoluzzi, Cristiano André Steffens, Viviane Aparecida Figueredo Oliveira Santos, Marcelo Alves Moreira, Bruno Jan Schramm Corrêa, Ricardo Trezzi Casa, Adelar Mantovani

**Affiliations:** 1 Instituto Federal de Santa Catarina, Câmpus Urupema, Urupema 88625-000, Brazil; 2 Centro de Ciências Agroveterinárias, Universidade do Estado de Santa Catarina, Florianópolis 88520-000, Brazil; roseli.bortoluzzi@udesc.br (R.L.d.C.B.); cristiano.steffens@udesc.br (C.A.S.); santos.vivioliveira@gmail.com (V.A.F.O.S.); marcelo.moreira@udesc.br (M.A.M.); brschramm@gmail.com (B.J.S.C.); ricardo.casa@udesc.br (R.T.C.); adelar.mantovani@udesc.br (A.M.)

**Keywords:** terpenes, natural fungicide, sustainable agriculture, Atlantic Forest

## Abstract

This study aimed to identify the volatile organic compounds (VOCs) present in the essential oil (EO) of *Schinus lentiscifolia* and to evaluate the effect of chitosan coatings (1%) enriched with EO of *S. lentiscifolia* (1000, 2000, and 4000 mg L^−1^) on the control of *Penicillium* sp. and on the quality of ‘Fuji’ apples. The EO was extracted from *S. lentiscifolia* collected in the municipality of Lages, Santa Catarina State, Brazil, in March, May, and November 2022. The antifungal activity of *S. lentiscifolia* EO against *Penicillium* sp. was evaluated in vitro. Apples were stored under refrigerated conditions (0 ± 0.5 °C; 90 ± 5% RH) for 30 days and subsequently under ambient conditions (23 ± 3 °C; 70 ± 5% RH) for 5 days. A total of 14 VOCs were identified in the EO of *S. lentiscifolia*, with the monoterpenes *β*-pinene (34.68%) and *α*-pinene (30.61%) as the major compounds, followed by *γ*-terpinene (10.13%), camphene (9.66%), and *o*-cymene (7.14%). The application of chitosan coating with *S. lentiscifolia* EO (2000 mg L^−1^) reduced the severity of blue mold in ‘Fuji’ apples by 88.1% during refrigerated storage and by 69.2% under ambient conditions. Ethylene production by the apples was also reduced when treated with chitosan and EO. No influence of the treatments was observed on fruit quality attributes. The postharvest application of chitosan coatings combined with *S. lentiscifolia* EO reduces disease caused by *Penicillium* sp. in ‘Fuji’ apples without affecting fruit quality.

## 1. Introduction

Apple (*Malus domestica* Borkh.) is one of the most widely cultivated temperate fruits in the world. According to data from FAO [1], global apple production in 2023 was approximately 97 million tons, with China as the largest producer, accounting for more than 49 million tons. Brazil produced approximately 1,183,000 tons of apples in 2023 [1], with the states of Santa Catarina and Rio Grande do Sul taking a part with 96.88% of the national production [2]. The main cultivars produced in Santa Catarina in 2022 were ‘Fuji’ and ‘Gala’, representing 52.5% and 45.2% of the planted apple-growing areas, respectively [3].

Some of the main constraints affecting the apple production chain are postharvest diseases, particularly blue mold, a decay caused by *Penicillium* spp. [4]. Apples of the ‘Fuji’ cultivar are susceptible to this pathogen, which not only leads to a loss of commercial value of fresh fruits but is also a mycotoxin-producing fungus that can contaminate agro-industrial by-products, such as apple juice [4,5,6,7,8]. Effective management of postharvest diseases involves appropriate harvesting, handling, and transportation practices; storage under conditions that limit pathogen growth, reproduction, and spread; as well as chemical control using fungicides [9]. Some fungicides registered in Brazil for the control of blue mold in postharvest apples have imazalil, from the imidazole chemical group, and pyrimethanil, from the anilinopyrimidine group, as active ingredients [10]. However, the safety of fungicide products, even those that are registered, has been questioned, since these molecules can penetrate the fruit peel and be present in the fruit pulp, representing a potential risk to consumer health [11,12]. Studies have demonstrated the potential risk of accumulation of these molecules in animal tissues, as well as their possible involvement in neurological diseases, endocrine disruption, and some chronic diseases [13,14,15].

Considering the disadvantages associated with the use of synthetic fungicides, products of natural origin, such as chitosan, plant extracts, and essential oils (EOs), have also been the focus of studies for the management of postharvest fruit decay [16]. Several studies have demonstrated that the application of biofilms or edible coatings based on chitosan combined with EOs can promote the maintenance of fruit quality and reduce the severity and incidence of postharvest diseases, with minimal sensory alterations when appropriate doses are used [17,18,19,20,21,22,23]. In addition, these products generally exhibit low toxicity compared with industrial chemical fungicides, allowing for safer use [24].

Chitosan is a natural polysaccharide obtained primarily from the chitinous exoskeleton of marine arthropods, such as shrimp, crab, and lobster, and is considered a safe material for application in edible products [23,25]. Essential oils (EOs), in turn, are complex mixtures of volatile and lipophilic substances, mainly terpenes, derived from the secondary metabolism of plants, and may be present in leaves, fruits, flowers, and other plant tissues [26,27,28]. Brazil is among the top five in export volume value of EOs, mainly from *Citrus* sp. and *Eucalyptus* sp., despite the potential of native plant species from Brazilian biomes such as the Atlantic Forest [29,30]. Among the many native plant species that have been studied regarding the potential and characteristics of their essential oils (EOs), those belonging to the genus *Schinus* sp. stand out, including *S. terebinthifolia* Raddi, *S. molle* L., and *S. lentiscifolia* Marchand [30,31,32]. Commonly known as “aroeira-do-campo,” *S. lentiscifolia* is a species native to southern Brazil, as well as to Argentina, Paraguay, and Uruguay [33]. Some studies have attributed antimicrobial potential to extracts and EOs obtained from the leaves of this species [34,35,36]. However, studies remain scarce.

Considering the need to develop new strategy for the management of postharvest apple decay, as well as to expand knowledge on the potential use of essential oils (EOs) extracted from native Brazilian plants, this study aimed to identify the volatile organic compounds in the essential oil of *Schinus lentiscifolia* and to evaluate the effect of chitosan coatings (1%) combined with *S. lentiscifolia* EO (1000, 2000, and 4000 mg L^−1^) on the control of *Penicillium* sp. and on the quality of ‘Fuji’ apples.

## 2. Materials and Methods

### 2.1. Extraction and Chemical Characterization of S. lentiscifolia Essential Oil

For essential oil extraction, leaves of *S. lentiscifolia* were collected on 8 March, 31 May, and 25 November 2022, in the municipality of Lages, Santa Catarina State, Brazil (20°04′23.58″ S and 50°29′16.79″ W). The material was packed in black plastic bags with a capacity of 100 L and transported for approximately 1 h under uncontrolled temperature and humidity conditions to Herbário de Lages da Universidade do Estado de Santa Catarina (LUSC), at the Centro de Ciências Agroveterinárias (CAV) of Universidade do Estado de Santa Catarina (UDESC), where it was dried at 25.1 °C (±1.1) and relative humidity of 49.1% (±4.3) for 8 days, followed by storage in black plastic bags sealed hermetically.

EO extraction was performed by steam distillation using a Solab^®^ distiller, model SL-76/I. The extraction process for each sample lasted 5 h at a temperature of 115 °C. The EOs were stored in 10 mL amber glass vials, labeled according to the sample and collection date, and kept at room temperature protected from light [37]. After this process, for the purposes of this experiment, EOs extracted on the three dates mentioned above were mixed, and this mixture was also stored at room temperature, protected from light.

For the chemical characterization of the EO, initially, 0.2 g of EO from each sample was weighed and placed in a 40 mL vial. The vial was sealed with a screw cap fitted with a polytetrafluoroethylene (PTFE)/silicone septum to prevent leakage. The sample was maintained at 25 °C ± 1 °C for 30 min. After the system reached equilibrium (10 min), the headspace air of the vial, containing the volatile compounds of the oil, was collected using a 100 µL syringe (Sigma-Aldrich Co., A-series gas syringe, St. Loius, MO, USA), followed by injection of the sample into the chromatograph injector.

The compounds were separated on an Elite 5MS capillary column (30 m × 0.25 mm, 0.25 μm) using a gas chromatograph (PerkinElmer, model Clarus 680, PerkinElmer Inc. Waltham, MA, USA) coupled to a mass spectrometer (Perkin Elmer, model Clarus SQ8S, USA). The injector and detector temperatures were set at 230 °C, and the total run time was 12.5 min, with an initial oven temperature of 50 °C, followed by a heating ramp at a rate of 8 °C min^−1^ up to 110 °C, and subsequently 80 °C min^−1^ up to 270 °C. In addition, to improve peak separation, a split flow of 35 mL min^−1^ was applied. Helium was used as the carrier gas at a flow rate of 1 mL min^−1^.

Compound identification was based on spectra available in the National Institute of Standards and Technology (NIST) library, version 2.0, 2014. Compounds were identified solely by comparison of the experimental mass spectra with those from the NIST library and were thus considered identified. Based on retention times (RT), retention indices (RI) were calculated, and the values were compared with those reported in the literature for each compound.

### 2.2. Evaluation of the In Vitro Antifungal Potential of the EO

The pathogen *Penicillium* sp. was isolated from apples showing symptoms and signs of blue mold. Fungal identification at the genus level was performed through evaluations of colony characteristics as well as morphology of pathogen structures, such as conidiophores and conidia. The isolate was preserved and stored in the mycological collection of the Plant Pathology Laboratory at CAV/UDESC.

For the experiment, conidia of the previously isolated pathogen were collected using autoclaved water and Tween^®^, and, with the help of a Drigalski spatula, spread over the entire surface of Petri dishes containing PDA medium (Potato Dextrose Agar). These plates were incubated in a BOD chamber at 25 °C and 95% relative humidity for 7 days. After fungal growth and sporulation, 9 mm discs of medium containing mycelium and conidia of the pathogen were removed from these plates and transferred to new plates also containing PDA medium.

A 3 cm × 3 cm adhesive paper label impregnated with *S. lentiscifolia* EO at concentrations of 0, 1, 5, 10, 50, 100, 200, 300, 400, 500, and 1000 µL L^−1^ was fixed to the inner upper surface of each of these Petri dishes, with each concentration applied in 10 replicates.

The plates containing pure fungal cultures and EOs were closed and sealed with flexible thermoplastic film (Parafilm^®^) to allow the compounds present in the oils to come into contact with the fungal mycelia through volatilization. The plates were incubated for six days in a BOD chamber at 25 °C and 95% relative humidity, and fungal colonies were measured using a ruler every two days (48 h, 96 h, and 144 h). As a control, plates inoculated with *Penicillium* sp. received adhesive without EO. The entire procedure was carried out in triplicate.

Mycelial growth inhibition (MGI, %) for each evaluation day was calculated according to the following formula [37,38]:

$$IC\left(\%\right)=\frac{\left(Dc-Dt\right)}{Dc}\times 100$$

where:

$D_{c}$ is the colony diameter (mm) in the control treatment;

$D_{t}$ is the colony diameter (mm) in the treatments with essential oils.

### 2.3. Evaluation of the Antifungal Potential of Chitosan Coatings with S. lentiscifolia EO

‘Fuji’ apples were harvested from a commercial orchard located in the municipality of São Joaquim, SC, in May 2023. The apples were packed in plastic crates suitable for fruit transportation and transported for 2 h under uncontrolled temperature and humidity conditions to the laboratory, where they were subjected to the analysis of ripening attributes. The iodine–starch index was determined using a scale from 1 to 5, where index 1 indicates the maximum starch content and index 5 represents fully hydrolyzed starch. In addition, analyses of background skin color, titratable acidity, soluble solids, and flesh firmness were performed according to the methodologies described below in Section 2.4. The apples showed, on average, 14.8 °Brix, 0.35% titratable acidity (malic acid), and 78.8 N flesh firmness. Regarding skin color, the Hue angle (h°) value averaged 93.95.

Subsequently, the apples were stored in a cold chamber at 0 ± 2 °C and 90 ± 5% relative humidity until the experiment was established.

After sanitization in a 1% sodium hypochlorite solution, the apples were dried at room temperature for 8 h. For fruit inoculation, isolates of the pathogen *Penicillium* sp. were subcultured on Petri dishes and incubated in a BOD chamber with a 12 h photoperiod at 25 °C (±0.8) to obtain a conidial suspension. After sporulation of the isolates, conidia were collected using a Drigalski spatula and distilled water containing Tween^®^, and a suspension was prepared in distilled water. The conidial concentration was 1.87 × 10^6^ CFU mL^−1^, determined using a Neubauer chamber and an optical microscope.

To ensure pathogen inoculation, the fruits were perforated in the equatorial region at two equidistant points using an Extralab^®^ electronic texturometer equipped with a 2 mm diameter probe, with a perforation depth of 3 mm. Subsequently, the fruits were inoculated using a pipette by introducing 10 µL of the previously prepared conidial suspension into each wound. After inoculation, the fruits were kept at room temperature for 24 h before the application of treatments.

The chitosan coating with essential oils (EOs) was prepared by dissolving 10 g of this polymer in a 1% acetic acid solution (10 mL of acetic acid in 1000 mL of distilled water), ensuring complete dissolution. Subsequently, 1% glycerin and 1% Tween^®^ were added to the solution, whose volume was 1000 mL. The essential oil was then incorporated into the chitosan solution in appropriate amounts to obtain concentrations of 1000, 2000, and 4000 mg L^−1^. The EO–chitosan mixtures were then subjected to sonication using a Bandelin Sonoplus 2200^®^ ultrasonic homogenizer, equipped with an MS 72 probe, at a frequency of 20 kilohertz (kHz) in four cycles of 5 min each. Through this process, EO micelles were disrupted until a homogeneous solution was obtained.

After preparation of the solutions, they were applied by spraying until reaching the point of runoff on apples previously inoculated with *Penicillium* sp. using spray bottles [39]. The treatments were as follows: control (no application); 1% chitosan solution; and 1% chitosan solution + *S. lentiscifolia* EO at concentrations of 1000, 2000, and 4000 mg L^−1^.

The apples were then placed in a ventilated area to allow coating drying and improve its adhesion to the fruit surface for 24 h at room temperature [40]. Subsequently, the fruits were stored in a cold chamber (0 ± 0.5 °C; 90 ± 5% RH) for 30 days. Thereafter, the fruits were kept under ambient conditions (23 ± 3 °C/70 ± 5% RH) in a climate-controlled room for 5 days. Storage under ambient conditions was monitored using a portable digital thermo-hygrometer (Instrutherm^®^, model HTR-157).

Throughout the storage period and exposure under ambient conditions, blue mold severity was evaluated. The treatments were arranged in four replicates of 10 fruits each, totaling 40 fruits per treatment. Severity analysis was performed by measuring the diameter of lesions present on the fruits using a ruler on the first day of storage. Subsequently, lesion diameters were measured at the following intervals: 7th, 10th, 13th, 16th, 19th, 22nd, 25th, 27th, and 30th days of refrigerated storage (0 ± 0.5 °C/90 ± 5% RH). After this period, the fruits were maintained under ambient conditions (23 ± 3 °C; 70 ± 5% RH), and lesion diameters caused by *Penicillium* sp. were also measured daily under these conditions until the fifth day.

Using the lesion diameter data, the lesion area (mm^2^) of the fruits was calculated under both storage conditions, and the area under the disease progress curve (AUDPC) was also determined using the following formula [41,42]:

$$AUDPC=\frac{\Sigma (Si+Si+1)}{2(Ti+1-Ti)}$$

where:

AUDPC = area under the disease progress curve (severity);

$S_{i}$ = disease severity, mean lesion diameter at the ith observation;

$S_{i+1}$ = disease severity, mean lesion diameter at the (i + 1)th evaluation;

$T_{i}$ = time (days) at the ith observation;

$T_{i+1}$ = time (days) at the (i + 1)th evaluation.

### 2.4. Evaluation of the Effect of Coating Application on Fruit Ripening and Quality

Non-inoculated apples received the same treatments described previously and were stored under conditions identical to those of the inoculated apples. Each treatment was performed with four replicates of 20 fruits, totaling 80 fruits per treatment. Analyses of respiration rate, ethylene production, and flesh firmness were carried out on the first day and on the last day of storage under ambient conditions (5 days).

Titratable acidity (TA, % malic acid) values were obtained from a 5 mL juice sample extracted by processing the fruits in a centrifuge. This sample was diluted in 45 mL of distilled water and titrated with 0.1 N NaOH solution up to pH 8.1, using an automatic titrator TitroLine^®^ easy (SCHOTT Instruments, Mainz, Germany).

Soluble solids (SS, °Brix) were determined using a digital refractometer, model PR201α (Atago^®^, Tokyo, Japan), with an aliquot of juice obtained from fruit processing.

Skin color was evaluated in terms of “L” (lightness), chroma (C), and hue angle (h°) values using a colorimeter (Konica Minolta, Tokyo, Japan). Measurements were taken at two opposite points in the equatorial region of the fruits.

Flesh firmness (N) was determined in the equatorial region of the fruits, at two opposite surfaces, after removing a small portion of the epidermis, using an electronic penetrometer (GÜSS Manufacturing Ltd., Cape Town, South Africa) equipped with a 7.9 mm diameter probe.

Respiration rates (ηmol CO_2_ kg^−1^ s^−1^) and ethylene production rates (ηmol C_2_H_4_ kg^−1^ s^−1^) were quantified by gas chromatography, based on the analysis of CO_2_ produced by the fruits and accumulated in the container using an electronic gas analyzer (Oxicarb 6 model, Isolcell S.p.A., Laives, BZ, Italy). Fruits from each replicate were placed in 4.1 L containers with hermetic sealing. Respiration and ethylene production rates were determined from the difference in CO_2_ and C_2_H_4_ concentrations, respectively, inside the container immediately after sealing and after 30 min.

After this period, two samples of the headspace atmosphere of these containers were collected using a 1.0 mL plastic syringe and injected into a gas chromatograph (PerkinElmer^®^, model Clarus 580, PerkinElmer Inc., Shelton, CT, USA) equipped with a 3 m Porapak N^®^ column (80–100 mesh) and a flame ionization detector.

Volatile compounds from the fruits were isolated using the SPME technique. Analyses were performed on a GC–MS system (PerkinElmer Inc. Waltham, MA, USA), consisting of a gas chromatograph (Clarus 680 GC model) coupled to a mass spectrometer (Clarus SQ 85 model). In a 40 mL vial sealed with a screw cap and a PTFE-coated silicone septum (Agilent Technologies, Germany), 1 g of fruit sample, 1 g of NaCl, and 5 mL of deionized water were added. The sample was then subjected to pre-extraction incubation (equilibration) under agitation for 10 min at 30 ± 1 °C, after which the SPME fiber (CAR/PDMS), mounted on a holder, was exposed to the headspace of the sample for 25 min. Subsequently, the fiber was exposed for 10 min for desorption at 250 °C in the chromatograph injector.

The column used was HP-5, and helium was employed as the carrier gas at a flow rate of 1 mL min^−1^. The initial temperature was 30 °C for 5 min, followed by a heating ramp of 2 °C min^−1^ up to 120 °C, where it was held for 3 min, totaling a run time of 34 min. Compound identification was based on the chromatograph library, which indicates the probability of similarity of each compound with the data contained in the software. To confirm compound identification, 3 µL of a C7–C40 n-alkane standard was analyzed under the same conditions as the samples. The percentage of each compound was determined based on the area of the nearest n-alkane [43].

### 2.5. Statistical Design

The experiment was conducted in a completely randomized design. Data on AUDPC, titratable acidity, soluble solids, skin color, flesh firmness, and respiration and ethylene production rates were subjected to analysis of variance, and treatment means were compared using Tukey’s test (*p* < 0.05), with the help of SAS 9.0 software (SAS Institute, 2002). The means of mycelial growth inhibition (MGI, %) as a function of *S. lentiscifolia* EO concentrations were subjected to regression analysis.

## 3. Results and Discussion

### 3.1. Characterization of Volatile Organic Compounds of S. lentiscifolia Essential Oil and Its Effect on the Mycelial Growth of Penicillium sp. In Vitro

A total of 14 volatile organic compounds were identified in the essential oil (EO) of *S. lentiscifolia*, with the monoterpenes *β*-pinene (34.68%) and *α*-pinene (30.61%) as the major constituents, followed by *γ*-terpinene (10.13%), camphene (9.66%), and *o*-cymene (7.14%) (Table 1). There are few studies available on the essential oils of *S. lentiscifolia*. Danielli et al. [34] analyzed the chemical composition of EO extracted from leaves of *S. lentiscifolia* collected in Rio Grande do Sul, identifying 31 compounds, with *γ*-eudesmol (12.8%), *β*-eudesmol (10.2%), *α*-eudesmol (9.2%), elemol (10.5%), *β*-caryophyllene (9.9%), and α-pinene (8.2%) as the major constituents. Pawlowski et al. [35] identified 50 compounds in the EO from leaves of *S. lentiscifolia*, also collected in Rio Grande do Sul, with δ-cadinene (14.21%), limonene (8.14%), sabinene (5.08%), α-cadinol (4.91%), and α-pinene (4.80%) as the predominant compounds. The composition of essential oils from the same species may vary depending on the time of year of collection, edaphoclimatic conditions of the collection site, genetic variability, plant age and phenological stage, extraction method, and the analytical methodology used in chromatographic analysis, among other factors [44,45,46]. These factors explain the differences observed between the results reported by Danielli et al. [34] and Pawlowski et al. [35], as well as those obtained in the present study.

**Figure 1:**
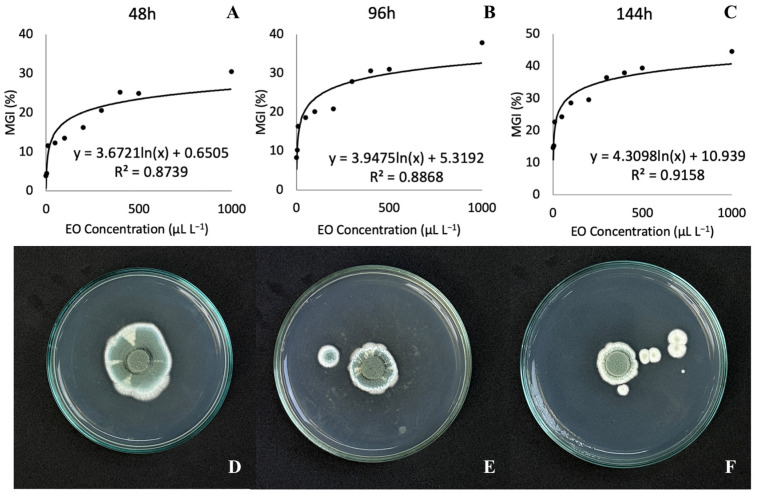
Dispersion of mean mycelial growth inhibition (MGI, %) of *Penicillium* sp. colonies in vitro treated with *Schinus lentiscifolia* essential oil (EO) and incubated for 48 h (**A**), 96 h (**B**), and 144 h (**C**) in a BOD chamber at 25 °C (±0.8) and 95% relative humidity (RH). *Penicillium* sp. colonies after 144 h of incubation: without EO application (**D**); treated with *S. lentiscifolia* EO at concentrations of 500 µL L^−1^ (**E**) and 1000 µL L^−1^ (**F**).

With regard to the in vitro mycelial growth inhibition (%) of *Penicillium* sp., the highest mean inhibition rates were 30% after 48 h and 38% after 96 h, both at the concentration of 1000 µL L^−1^; and 36%, 38%, 39%, and 44% after 144 h at EO concentrations of 300, 400, 500, and 1000 µL L^−1^, respectively (Figure 1). The essential oil of *S. lentiscifolia* promoted greater inhibition of mycelial growth with increasing concentration. However, complete inhibition was not achieved, and a stabilization of the inhibitory effect on mycelial growth was observed as the concentrations increased, which may indicate a limitation in the antifungal potential of this essential oil against *Penicillium* sp.

Essential oils generally exhibit antifungal potential due to their chemical characteristics. However, this potential varies according to the concentration and the compounds present in the oil, the method of application, and the population, species, and strain of the target microorganism [36,47]. The mechanisms of action of essential oils on fungal cells may involve, according to El Khetabi et al. [47], mitochondrial dysfunction with reduced ATP synthesis; interference with protein synthesis and cytoplasmic coagulation; inhibition of cell wall formation; damage to the cytoplasmic membrane, leading to increased permeability and leakage of cellular contents; and damage to membrane proteins.

Valková et al. [48] evaluated the antifungal activity of essential oils from mint (*Mentha* sp.), lavender (*Lavandula* sp.), and rosemary (*Rosmarinus officinalis*) against *Penicillium citrinum*, *P. crustosum*, and *P. expansum* in vitro, and observed differences in the inhibition of mycelial growth of the pathogens depending on the concentration and type of oil, as well as on the fungal species. Mint essential oil exhibited the greatest antifungal effect against all three pathogen species compared with the other essential oils tested.

Zulu et al. [49] compared the antifungal activity of 15 different commercial essential oils (EOs) against Penicillium digitatum. The authors [49] used EOs of patchouli (*Pogostemon cablin*), chamomile (*Matricaria chamomilla*), cedar (*Cedrus deodara*), rose (*Rosa damascena*), crown daisy (*Glebionis coronaria*), dill (*Anethum graveolens*), vetiver (*Chrysopogon zizanioides*), lemon balm (Melissa officinalis), niaouli (*Melaleuca quinquenervia* and *M. alternifolia*), oregano (*Origanum vulgare*), thyme (*Thymus vulgaris*), vanilla (*Vanilla fragrans*), ylang-ylang (*Cananga odorata*), and cinnamon (*Cinnamomum verum*). The authors [50] observed that the antifungal activity was dose-dependent, with higher doses resulting in greater effects. Cedar, rose, niaouli, and thyme EOs exhibited insignificant effects on mycelial growth and sporulation inhibition. The best in vitro result was observed with cinnamon EO application, showing 100% inhibition of sporulation and germination. Cinnamon EO was the only treatment that completely inhibited mycelial growth at a concentration of 1.25 μL/mL and conidial germination at an EC50 value of 0.424 μL/mL. Mycelial growth was completely inhibited by oregano and ylang-ylang EOs at a concentration of 5 μL/mL. The authors also evaluated some effects of the EOs on fungal structures. Application of vetiver and patchouli EOs caused hyphal constriction and inhibition of conidial germination. Cinnamon and chamomile EOs also affected conidia, causing severe morphological deformation and cellular collapse. Oregano EO also induced morphological alterations in the hyphae. Furthermore, changes in pigment accumulation in fungal structures were observed in response to all tested EOs.

Bahri et al. [50] tested the antifungal capacity of essential oils against *Penicillium expansum* in vitro and observed a reduction in mycelial growth of up to 64% with the application of clove essential oil (*Syzygium aromaticum*) incorporated into PDA medium, as well as a reduction of up to 97% in conidial production. In contrast, the application of mint essential oil (*Mentha pulegium*) resulted in lower inhibition of mycelial growth, up to 13.6%, although colonies treated with this oil showed an 86% reduction in conidial production.

The studies cited above highlight both the antifungal potential of essential oils and the variability of this potential depending on the type of oil, its concentration, chemical composition, the target pathogen, and the methodology used for application. Some studies have reported the antimicrobial potential of essential oils from *S. lentiscifolia*. Danielli et al. [34] and Gehrke et al. [36] observed antifungal and antibacterial activity in essential oils of *S. lentiscifolia*. However, the composition of the essential oils used in those studies differs from that found in the present work, in which the main compounds were α-pinene and β-pinene, as previously discussed. There are still no studies evaluating the effect of *S. lentiscifolia* essential oil on *Penicillium* sp.; however, the compounds α-pinene and β-pinene have shown promising activity against this pathogen. Valková et al. [48] evaluated the antifungal effect of fir essential oil (*Abies alba*), a species from the family Pinaceae, whose main compounds were α-pinene (25.2%), β-pinene (18.3%), and α-limonene (18.1%). The authors [48] observed antifungal activity of this essential oil against species of *Penicillium* sp., including *P. expansum*, a pathogen associated with blue mold in apples, at concentrations of 125, 250, and 500 µL L^−1^.

### 3.2. Effect of In Vivo Application of Chitosan Coating with S. lentiscifolia Essential Oil on Blue Mold Severity (Penicillium sp.) in ‘Fuji’ Apples

**Figure 2:**
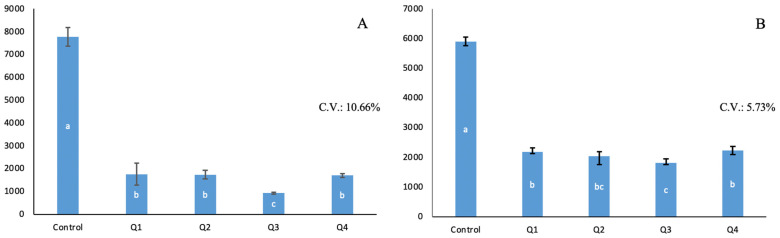
Comparison of the mean area under the disease progress curve (AUDPC) caused by *Penicillium* sp. for each treatment: chitosan (Q1) and chitosan supplemented with *Schinus lentiscifolia* essential oil (EO) at concentrations of 1000 mg L^−1^ (Q2), 2000 mg L^−1^ (Q3), and 4000 mg L^−1^ (Q4), compared with the control. Evaluations were performed during 30 days of refrigerated storage (0 ± 0.5 °C and 90 ± 5% relative humidity (RH)) of ‘Fuji’ apples (**A**) and during storage under ambient conditions (23 ± 3 °C and 70 ± 5% RH) (**B**). Means followed by the same letters are not significantly different according to Tukey’s test (*p* < 0.05).

During refrigerated storage, a reduction in blue mold severity was observed in apples subjected to all treatments compared with untreated fruit, considering the area under the disease progress curve (AUDPC). The application of chitosan biofilm without the addition of *S. lentiscifolia* essential oil reduced disease severity by 77.4%. In contrast, the application of chitosan coating with *S. lentiscifolia* essential oil at a concentration of 2000 mg L^−1^ resulted in an 88.1% reduction in disease severity. In apples treated with chitosan combined with essential oil at concentrations of 1000 and 4000 mg L^−1^, reductions of 77.6% and 78.1%, respectively, were observed, with no statistical difference compared with the treatment with chitosan alone according to Tukey’s test (*p* < 0.05) (Figure 2A).

Throughout the period under ambient conditions following refrigerated storage, an increase in disease severity was observed in all treatments, highlighting the influence of temperature on disease progression. However, once again, the AUDPC values of fruits treated with chitosan coatings, with or without essential oils, were markedly lower than those of untreated fruits. Fruits treated with chitosan coating combined with *S. lentiscifolia* essential oil at 2000 mg L^−1^ exhibited an AUDPC 69.2% lower than the control, differing statistically from the other treatments according to Tukey’s test (*p* < 0.05). The other treatments promoted reductions of 62.2% (1% chitosan), 62.0% (1% chitosan + *S. lentiscifolia* EO at 4000 mg L^−1^), and 65.4% (1% chitosan + *S. lentiscifolia* EO at 1000 mg L^−1^) (Figure 2B).

**Figure 3:**
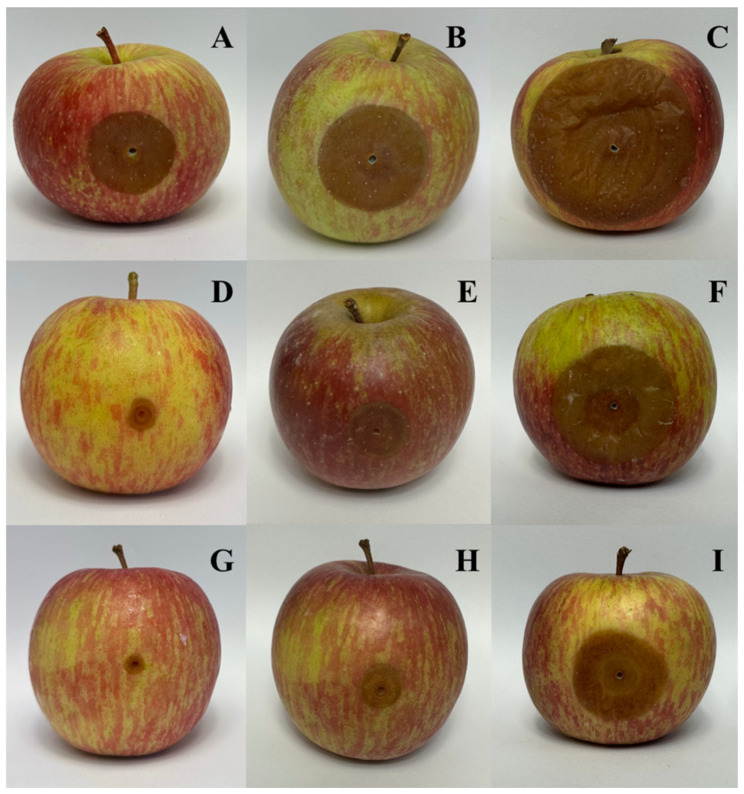
Apples inoculated with *Penicillium* sp.: control (**A**–**C**); treated with 1% chitosan (**D**–**F**); 1% chitosan + *S. lentiscifolia* essential oil at a concentration of 2000 mg L^−1^ (**G**–**I**). Evaluations were performed on the 19th (**A**,**D**,**G**) and 30th (**B**,**E**,**H**) days of refrigerated storage and on the 5th day under ambient conditions (**C**,**F**,**I**).

Figure 3 shows untreated apples (A, B, and C), apples treated with 1% chitosan (D, E, and F), and apples treated with chitosan combined with *S. lentiscifolia* essential oil at a concentration of 2000 mg L^−1^ (G, H, and I), evaluated after 19 and 30 days of refrigerated storage and after an additional five days under ambient conditions.

The antifungal activity of chitosan coatings in postharvest fruit has been extensively studied. Romanazzi et al. [51] analyzed several studies on the antimicrobial properties of chitosan coatings in postharvest systems and attributed the effectiveness of this polymer to three main factors: its direct antimicrobial activity, the formation of a biofilm acting as a physical barrier on the fruit surface, and its elicitor or plant defense-inducing effect. The direct antimicrobial activity of chitosan is associated with the inhibition of fungal spore germination and the reduction in mycelial growth in already infected fruits, including both quiescent and active infections. The application of chitosan also affects the fruit’s own defense mechanisms, and its influence on enzymes such as chitinase is well documented. In addition, the formation of a chitosan biofilm on the fruit surface affects gas exchange with the atmosphere, reducing fruit respiration and consequently decreasing the effects of fungal pathogenicity. However, the effectiveness of chitosan depends on several factors, such as polymer concentration, plant species, and pathogen species [17,51,52,53,54].

These studies support the results obtained with the application of chitosan coating combined with *S. lentiscifolia* essential oil at 2000 mg L^−1^. However, at the concentration of 4000 mg L^−1^ of the oil, no statistical difference in blue mold severity (AUDPC) was observed compared with the application of chitosan without essential oil. Increasing the concentration of essential oil, depending on its chemical characteristics and the plant product, may induce phytotoxicity. Di Francesco et al. [55] evaluated the antifungal efficacy of essential oils of thyme (*T. vulgaris*), lavender (*Lavandula angustifolia*), and rosemary (*Rosmarinus officinalis*) against several pathogens, including *P. expansum*, in postharvest apples. The authors [55] observed a reduction in disease severity after 7 days of storage at 20 °C, including blue mold, in inoculated apples. However, greater antifungal efficacy was observed at lower concentrations of lavender essential oil. Regarding thyme essential oil, reduced antifungal efficacy at higher concentrations was associated with probable phytotoxic effects [55].

### 3.3. Effect of Treatments with Chitosan Coatings and S. lentiscifolia Essential Oils on the Postharvest Quality of ‘Fuji’ Apples

**Figure 4:**
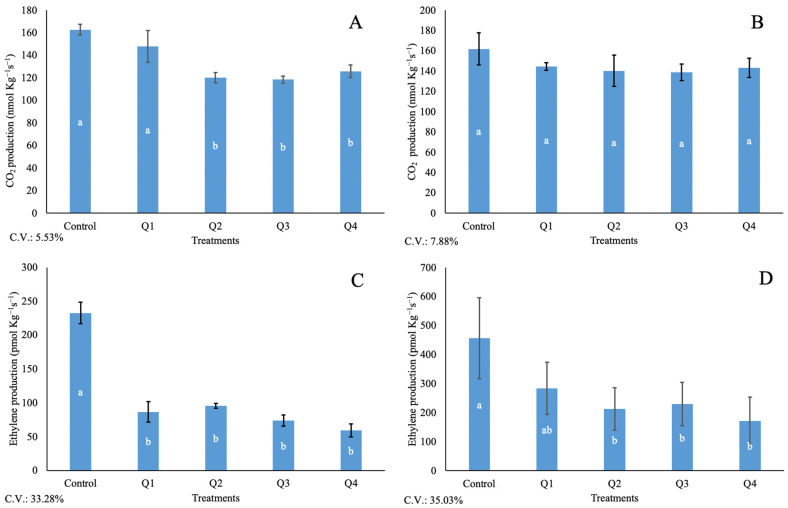
Respiratory rate of ‘Fuji’ apples coated with a 1% chitosan-based biofilm (Q1), with and without the addition of *S. lentiscifolia* essential oil at concentrations of 1000 (Q2), 2000 (Q3), and 4000 mg L^−1^ (Q4), compared with the control, after 30 days of refrigerated storage (0 ± 0.5 °C; 90 ± 5% RH) (**A**) and after 5 days of storage under ambient conditions (23 ± 3 °C; 70 ± 5% RH) (**B**). Ethylene production under the same treatments described above after 30 days of refrigerated storage 0 ± 0.5 °C; 90 ± 5% RH) (**C**) and after 5 days under ambient conditions (23 ± 3 °C; 70 ± 5% RH) (**D**). Means followed by the same letters do not differ significantly according to Tukey’s test (*p* < 0.05).

The respiration rate, after 30 days of storage and at chamber removal, was lower in fruits treated with 1% chitosan and essential oil at concentrations of 1000, 2000, and 4000 mg L^−1^ compared with untreated fruits (Figure 4A). However, after the storage period followed by exposure to ambient conditions, the respiration rates of apples did not differ among treatments (Figure 4B). Several studies have demonstrated the influence of coatings containing chitosan and the application of essential oils on respiration rates and ethylene production in postharvest fruits. Shah et al. [56] observed lower respiration rates in ‘Royal Gala’ apples treated with chitosan coatings. Furthermore, during storage, coated fruits did not exhibit a climacteric peak and, therefore, the authors attributed the reduction in respiration to the barrier effect promoted by the coating, which restricted gas exchange.

At chamber removal, fruits coated with 1% chitosan, with or without the addition of essential oil, did not differ among themselves in terms of ethylene production rate, but showed lower values than untreated fruits (Figure 4C). After an additional 5 days under ambient conditions, only the treatments combining chitosan with essential oil, at all evaluated concentrations, reduced the ethylene production rate compared with the control (Figure 4D). Malekipoor et al. [57] reported that treatments with lemon (*Citrus* sp.) and cinnamon (*C. verum*) essential oils reduced ethylene production in ‘Granny Smith’ and ‘Cripps Pink’ apples, while maintaining quality parameters in both cultivars stored under controlled atmosphere conditions. According to the authors, the delay in the ripening process of apples may be attributed to competition for ethylene binding sites in plant cells by the compounds (S)-(-)-limonene and trans-cinnamaldehyde, which are the main constituents of lemon and cinnamon essential oils, respectively [57].

No differences were observed among treatments for apple skin color attributes, both at the end of refrigerated storage and after an additional 5 days under ambient conditions, nor for soluble solids, titratable acidity, and flesh firmness (Table 2). When compared with the fruit maturity characteristics determined prior to the beginning of the experiment, slight changes were observed after 30 days of cold storage followed by 5 days under ambient conditions, including a decrease in hue angle (h°), an increase in soluble solids (SS), and a slight reduction in titratable acidity (TA), indicating the progression of the ripening process. However, these changes were not pronounced, likely due to the relatively short storage period, considering that ‘Fuji’ apples can be stored for more than 3 months under appropriate conditions [58,59]. The results obtained indicate that treatments with chitosan coatings combined with *S. lentiscifolia* essential oil, at the evaluated concentrations, did not affect apple quality. However, as previously discussed, essential oil concentrations, application methods, and the chitosan concentration in the coating may influence fruit quality and may even cause phytotoxic symptoms [56].

Arrarte et al. [60] observed that chitosan coating in ‘Red Delicious’ apples under refrigerated storage resulted in reduced loss of flesh firmness. In addition, no significant differences were observed by the authors in soluble solids content or skin color. Long et al. [61] reported that chitosan coating (0.5%) combined with fennel essential oil (1%) significantly inhibited respiration rate, maintained fruit firmness, soluble solids content, titratable acidity, and skin gloss of apples during storage. Vieira et al. [62] also observed no influence, at the end of storage, of treatments with essential oils of rosemary (*Rosmarinus officinalis* L.), cinnamon (*Cinnamomum zeylacium* Blume), citronella (*Cymbopogon winterianus* Jowitt), and clove (*Syzygium aromaticum* L.) on soluble solids, titratable acidity, flesh firmness, and skin color of ‘Fuji’ apples. Heinzen et al. [17] likewise reported no effect of the application of chitosan and essential oils of citronella (*Cymbopogon winterianus*), copaiba (*Copaifera langsdorffii*), clove (*S. aromaticum*), and cinnamon (*Cinnamomum zeylacium*) on the postharvest quality of ‘Fuji’ apples.

The results obtained in the present study indicate that the use of chitosan coatings supplemented with *S. lentiscifolia* essential oil did not affect the ripening and senescence processes during storage. However, further studies are required to evaluate different concentrations of essential oils and chitosan in the coating solution. In addition, longer storage periods may better elucidate the potential effects of these treatments on fruit quality attributes.

### 3.4. Volatile Compound Profile

A total of 36 volatile organic compounds were identified, including 5 alcohols, 25 esters, 4 aldehydes, and 2 terpenes (Table 3). The compounds found at the highest concentrations in all treatments, including the control, were hexanal, (E)-2-hexenal, 1-hexanol, and 2-methyl-1-butyl acetate. Additionally, the following compounds were identified at lower concentrations: *n*-butyl acetate, 2-methyl-1-butanol, hexyl ethanoate, ethyl hexanol, and α-farnesene. The ester octyl butanoate was identified in the pulp of treated apples at area percentages lower than 0.2%, but was not detected in the control group.

The compounds 2-methyl-1-butyl acetate and hexyl ethanoate were identified in all treatments and are important contributors to the aroma profile of ‘Fuji’ apples [17,37,63,64].

The compounds originating from the essential oil of *S. lentiscifolia*, such as *β*-pinene, α-pinene, *γ* -terpinene, camphene, cubebol, among others, were not detected in any of the treatments. Heinzen et al. [17], when treating ‘Fuji’ apples with chitosan coatings combined with essential oils of citronella, copaiba, clove, and cinnamon, also did not identify components of the evaluated essential oils in the volatile profile of the fruit pulp. The authors attributed this result to the method of essential oil application, i.e., incorporation into a chitosan solution, which reduces the risk of compound migration from the fruit surface to the pulp.

## 4. Conclusions

A total of 14 volatile organic compounds (VOCs) were identified in the essential oil of *S. lentiscifolia*, with the monoterpenes *β*-pinene and *α*-pinene as the major constituents, followed by *γ*-terpinene, camphene, and *o*-cymene. The antifungal activity of EO can be attributed to the antifungal activity of the compounds that it consists of.

The application of chitosan coatings supplemented with *S. lentiscifolia* essential oil, especially at the concentration of 2000 mg L^−1^, reduced the severity of blue mold caused by *Penicillium* sp. during the storage of ‘Fuji’ apples, without negatively affecting fruit quality attributes under the tested storage conditions and duration. The application of chitosan coatings supplemented with *S. lentiscifolia* essential oil shows potential for use in the postharvest management of blue mold. This is a promising strategy for managing postharvest diseases in apples and, with further research, it has the potential to be commercialized. Results obtained in this study also recommend this strategy for managing other apple species and other fruits.

## Figures and Tables

**Table 1:** Volatile organic compounds identified in the essential oil of *Schinus lentiscifolia*.

No.	Compound ^1^	RT	RI	EO (%)
1	Tricyclene	4.56	919	1.86 ± 0.16
2	*α*-Pinene	4.72	930	30.61 ± 0.70
3	Camphene	5.00	948	9.66 ± 0.16
4	*γ*-Terpinene	5.35	972	10.13 ± 0.29
5	*β*-Pinene	5.45	978	34.68 ± 0.34
6	*β*-Myrcene	5.60	988	0.51 ± 0.04
7	*o*-Cymene	6.23	1026	7.14 ± 0.42
8	D-Limonene	6.32	1031	3.61 ± 0.14
9	Eucalyptol	6.39	1035	0.27 ± 0.05
10	*cis*-Muurola-3,5-diene	9.31	1370	0.12 ± 0.01
11	(−)-*β*-Bourbonene	9.34	1379	0.54 ± 0.02
12	Isocaryophyllene	9.45	1414	0.64 ± 0.09
13	α-Humulene	9.55	1453	0.14 ± 0.03
14	Cubebol	9.60	1474	0.09 ± 0.04

RT: retention time in the column. RI: retention index calculated based on the RT value. ^1^ NIST. National Institute of Standards and Technology. Mass Spectral Search Program for the NIST/EPA/NIH Mass Spectral Library, version 2.0. 2014.

**Table 2:** Peel color attributes (L, C, and h°), soluble solids (SS), titratable acidity (TA), and flesh firmness of ‘Fuji’ apples coated with 1% chitosan (Q1), with or without the addition of *S. lentiscifolia* essential oil at concentrations of 1000 (Q2), 2000 (Q3), and 4000 mg L^−1^ (Q4), compared with the control, after 30 days of refrigerated storage (removal from cold chamber—day 0 in the table) (0 ± 0.5 °C and 90 ± 5% RH) plus 5 days (day 5 in the table) under ambient conditions (23 ± 3 °C; 70 ± 5% RH).

	Day 0			Day 5				Day 5	
Treatments	L	C	*h*°	L	C	*h*°	SS (°Brix)	TA (% Malic Acid)	Flesh Firmness (N)
Control	67.30 ns	36.1 ns	90.04 ns	68.48 ns	39.85 ns	90.12 ns	15.65 ns	0.32 ns	76.4 ns
Q1	66.76	36.48	88.40	67.57	37.73	88.00	15.93	0.35	78.9
Q2	67.39	36.17	90.80	66.97	36.98	90.16	16.23	0.35	79.5
Q3	66.72	35.37	88.05	67.56	37.03	89.41	15.93	0.31	78.9
Q4	64.58	35.24	85.16	65.78	35.88	89.23	16.08	0.33	77.4
C.V. (%):	2.04	1.97	2.95	2.36	2.55	3.28	3.0	2.3	2.6

Note: ns: means within a column are not significantly different according to Tukey’s test (*p* < 0.05); C.V.: coefficient of variation.

**Table 3:** Profile of volatile compounds identified in ‘Fuji’ apples coated with 1% chitosan, with and without the addition of *S. lentiscifolia* essential oil at concentrations of 1000, 2000, and 4000 mg L^−1^, compared with the control, after 30 days of refrigerated storage (removal from cold chamber) (0 ± 0.5 °C and 90 ± 5% RH) plus 5 days under ambient conditions (23 ± 3 °C; 70 ± 5% RH).

N°	Compound	Control	Q1	Q2	Q3	Q4	Class
1	(R)-2-Butanol	0.34 ± 0.10	0.70 ± 0.06	1.18 ± 0.32	1.22 ± 0.15	1.87 ± 2.85	Alcohol
2	1-Butanol	2.37 ± 0.49	1.64 ± 0.43	2.05 ± 0.09	2.56 ± 0.27	2.04 ± 1.93	Alcohol
3	1-Butanol, 2-methyl-	4.34 ± 1.59	3.01 ± 0.69	3.28 ± 0.21	3.70 ± 0.37	2.83 ± 1.90	Alcohol
4	cis-2-Hexenol	0.70 ± 0.33	0.40 ± 0.06	0.47 ± 0.15	0.61 ± 0.16	0.12 ± 0.25	Alcohol
5	1-Hexanol	10.51 ± 1.42	8.34 ± 1.03	8.46 ± 1.88	8.03 ± 3.04	6.72 ± 3.09	Alcohol
6	Ethylhexanol	5.25 ± 1.04	5.46 ± 0.24	4.57 ± 0.40	5.06 ± 0.36	6.46 ± 1.32	Alcohol
7	Ethyl acetate	0.40 ± 0.11	1.82 ± 1.39	3.40 ± 0.50	2.08 ± 0.79	1.49 ± 1.67	Ester
8	Ethyl propanoate	0.45 ± 0.17	0.95 ± 0.93	1.54 ± 0.38	0.77 ± 0.30	0.52 ± 0.55	Ester
9	n-Propyl acetate	0.86 ± 0.32	0.69 ± 0.27	1.01 ± 0.78	0.49 ± 0.15	0.36 ± 0.28	Ester
10	Isobutyl acetate	0.18 ± 0.05	0.35 ± 0.11	0.21 ± 0.14	0.25 ± 0.04	0.28 ± 0.21	Ester
11	Ethyl butanoate	1.85 ± 0.27	3.55 ± 1.06	3.96 ± 0.34	3.34 ± 0.75	2.04 ± 1.56	Ester
12	Propyl propanoate	1.02 ± 0.20	0.41 ± 0.28	0.48 ± 0.04	0.44 ± 0.13	2.11 ± 3.58	Ester
13	n-Butyl acetate	5.82 ± 0.41	8.01 ± 1.54	6.34 ± 0.65	6.67 ± 0.49	4.46 ± 2.76	Ester
14	2-Methyl-1-butyl acetate	8.19 ± 1.19	7.76 ± 2.33	8.71 ± 0.97	7.42 ± 1.64	5.83 ± 3.97	Ester
15	n-Propyl butanoate	2.71 ± 0.38	2.43 ± 0.32	1.85 ± 0.34	1.84 ± 0.26	1.27 ± 0.26	Ester
16	n-Butyl propanoate	1.92 ± 0.42	1.89 ± 0.24	1.31 ± 0.15	1.47 ± 0.11	1.38 ± 0.17	Ester
17	Pentyl acetate	0.69 ± 0.12	1.03 ± 0.09	0.62 ± 0.03	0.70 ± 0.08	0.71 ± 0.06	Ester
18	n-Propyl 2-methylbutanoate	1.96 ± 0.27	1.15 ± 0.16	0.98 ± 0.20	0.78 ± 0.23	0.61 ± 0.15	Ester
19	2-Methylbutyl propanoate	0.36 ± 0.02	0.25 ± 0.05	0.21 ± 0.01	0.20 ± 0.03	0.23 ± 0.04	Ester
20	Butyl butanoate	2.96 ± 0.69	3.51 ± 0.60	3.01 ± 0.41	3.65 ± 0.38	4.11 ± 0.74	Ester
21	Ethyl hexanoate	0.72 ± 0.18	1.98 ± 0.80	1.70 ± 0.33	1.13 ± 0.28	1.26 ± 0.50	Ester
22	Hexyl ethanoate	4.73 ± 1.35	5.83 ± 0.92	5.49 ± 1.09	5.03 ± 1.55	6.53 ± 0.75	Ester
23	Butyl 2-methylbutanoate	3.09 ± 0.47	2.46 ± 0.55	1.93 ± 0.29	2.11 ± 0.26	2.63 ± 0.42	Ester
24	2-Methylbutyl butanoate	0.29 ± 0.07	0.30 ± 0.07	0.26 ± 0.04	0.28 ± 0.05	0.44 ± 0.08	Ester
25	Propyl hexanoate	0.56 ± 0.23	0.36 ± 0.08	0.29 ± 0.03	0.26 ± 0.03	0.24 ± 0.05	Ester
26	Hexyl propanoate	0.79 ± 0.26	0.75 ± 0.13	0.58 ± 0.06	0.64 ± 0.18	0.97 ± 0.27	Ester
27	Octyl butanoate	ND	0.09 ± 0.02	0.07 ± 0.005	0.09 ± 0.02	0.25 ± 0.08	Ester
28	Hexyl butanoate	2.16 ± 0.69	2.35 ± 0.68	2.01 ± 0.36	2.63 ± 0.63	3.82 ± 1.10	Ester
29	Hexyl 2-methylbutanoate	4.00 ± 0.78	2.41 ± 0.72	1.88 ± 0.20	2.32 ± 0.35	3.54 ± 0.62	Ester
30	Hexyl hexanoate	0.42 ± 0.26	0.20 ± 0.06	0.15 ± 0.03	0.18 ± 0.03	0.26 ± 0.03	Ester
31	2,2,4-Trimethyl-1,3-pentanediol diisobutyrate	2.96 ± 1.28	1.23 ± 0.32	2.92 ± 1.25	1.66 ± 0.41	1.34 ± 0.59	Ester
32	Hexanal	12.11 ± 3.39	10.35 ± 2.21	11.00 ± 0.67	15.59 ± 4.86	10.79 ± 5.75	Aldehyde
33	(E)-2-Hexenal	10.73 ± 7.61	14.76 ± 2.16	15.16 ± 2.14	13.66 ± 1.88	17.19 ± 2.91	Aldehyde
34	Benzaldehyde	0.67 ± 1.17	0.16 ± 0.19	0.04 ± 0.02	0.05 ± 0.01	0.04 ± 0.04	Aldehyde
35	Decanal	0.03 ± 0.005	0.05 ± 0.03	0.06 ± 0.002	0.02 ± 0.03	0.09 ± 0.08	Aldehyde
36	α-Farnesene	3.99 ± 1.91	3.34 ± 0.86	2.66 ± 0.61	2.78 ± 1.65	5.01 ± 0.78	Terpene

Note: Data expressed as mean ± standard deviation. ND: not detected. Source: PubChem Compound Summary (https://pubchem.ncbi.nlm.nih.gov/compound/), accessed on 10 November 2023.

## Data Availability

The original contributions presented in this study are included in the article. Further inquiries can be directed to the corresponding author.
